# *N*-(5-Cyano­nonan-5-yl)benzamide

**DOI:** 10.1107/S2414314623006399

**Published:** 2023-07-28

**Authors:** Xueqing Song, William Li

**Affiliations:** a University of the District of Columbia, Chemistry, 4200 Connecticut Avenue, NW, Washington DC, 20008, USA; Howard University, USA

**Keywords:** crystal structure, hydrogen bonding, strecker reaction, amino acid synthesis

## Abstract

*N*-(5-Cyano­nonan-5-yl)benzamide, C_17_H_24_N_2_O, synthesized from the reaction between benzoyl chloride and 2-amino-2-butyl­hexa­nenitrile, is an important inter­mediate in amino acid synthesis. Inter­molecular N—H⋯O and C—H⋯O hydrogen bonds with N⋯O and C⋯O distances of 3.083 (2) and 3.304 (2) Å, respectively, link adjacent mol­ecules into chains along the *a* axis. The dihedral angle between the mean plane of the phenyl group and the plane of the amide group is 19.504 (4)°.

## Abstract 2

The title compound crystallizes in the ortho­rhom­bic space group *Pbca* with eight formula units per unit cell. The N—H group forms an inter­molecular N—H⋯O hydrogen bond to the amide carbonyl O atom, generating chains.

## Graphical abstract

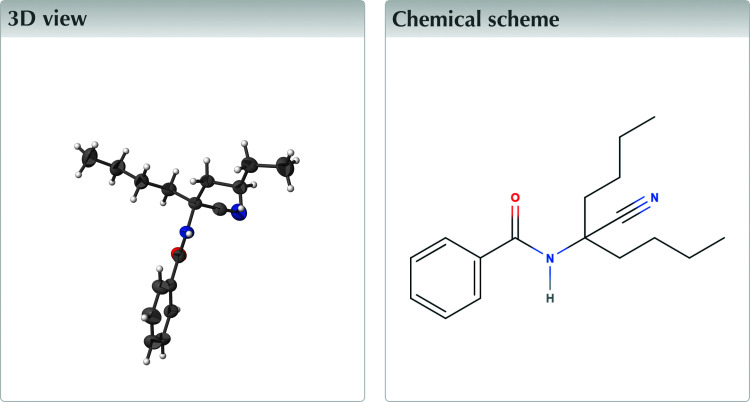

## Structure description

The title compound was synthesized from the reaction between 2-amino-2-butyl­hexa­ne­nitrile and benzoyl chloride, and is an important inter­mediate in amino acid synthesis. Shu *et al.* (2008 ▸) reported that a benzamide was an inter­mediate in their five-step synthesis of Fmoc-α-methyl­valine (Fmoc is the fluorenyl­meth­oxy­carbonyl protecting group). Paventi *et al.* (1987 ▸) found that the benzoyl group in the mol­ecule had assisted the hydrolysis of the nitrile in the acid hydrolysis of benzoyl­amino­nitrile to afford an α-amino acid. Some amino­nitriles were difficult to convert into α-amino acids without introducing a benzoyl group. An oxazoline inter­mediate was proposed to ease the acid hydrolysis of the nitrile in 2-benzamido­adamantane-2-carbo­nitrile.

**Figure 1:**
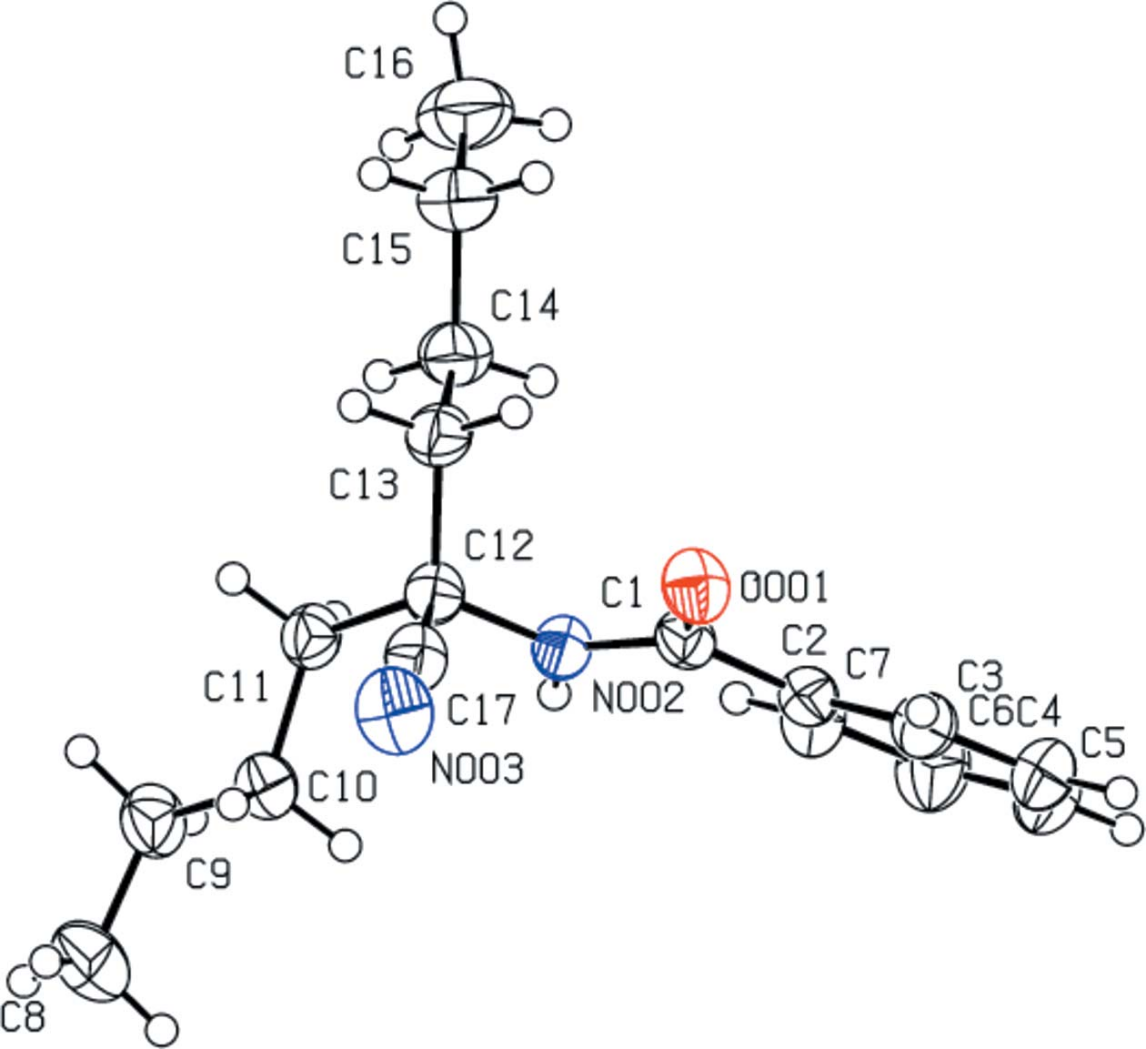
The mol­ecular structure of the title compound, showing the atom labeling. Displacement ellipsoids are drawn at the 50% probability level.

**Figure 2:**
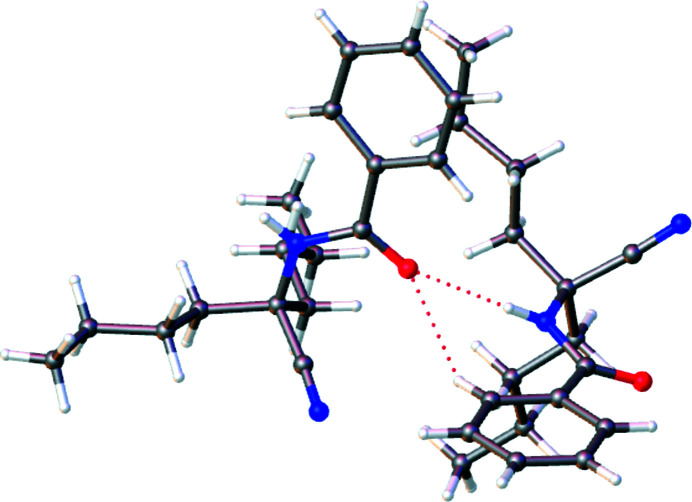
Inter­molecular N—H⋯O and N—H⋯O hydrogen bonds.

In the crystal of the title compound (Fig. 1 ▸), inter­molecular N—H⋯O and C—H⋯O hydrogen bonds with N⋯O and C⋯O distances of 3.083 (2) and 3.304 (2) Å, respectively, link adjacent mol­ecules into chains along the *a* axis (Table 1 ▸ and Figs. 2 ▸ and 3 ▸). The dihedral angle between the mean plane of the phenyl group and the plane of the amide O1/C1/N1/C12 group (r.m.s. deviation 0.002 Å) is 19.504 (4)°.

## Synthesis and crystallization

A two-step procedure was used to synthesize *N*-(5-cyano­nonan-5-yl)benzamide. The first step was the Strecker synthesis using nonan-5-one, ammonia, ammonium chloride and NaCN as starting materials to afford 2-amino-2-butyl­hexa­nenitrile. The second step was the reaction between 2-amino-2-butyl­hexa­nenitrile and benzoyl chloride in an aqueous solution of sodium bicarbonate to afford crude *N*-(5-cyano­nonan-5-yl)benzamide. This was then purified *via* column chromatography, and slow evaporation of a dilute solution in ethyl acetate afforded a needle-like crystal (m.p. 383–385 K).

^1^H NMR (CDCl_3_, ppm): δ 7.835–7.707 (2H, *m*), 7.621–7.526 (1H, *m*), 7.525–7.406 (2H, *m*), 6.145–5.982 (1H, *s*), 2.231–2.205 (4H, *m*), 1.656–1.339 (8H, *m*), 1.038–0.867 (6H, *m*). ^13^C NMR (CDCl_3_, ppm): δ 166.7, 133.8, 132.0, 128.9, 127.0, 119.9, 55.4, 36.3, 26.4, 22.6, 13.7.

## Refinement

The crystal data, data collection and structure refinement details are summarized in Table 2 ▸. The amide H atom was refined isotropically. All other H atoms were refined with isotropic displacement parameters, calculated as *U*_iso_(H) = 1.5*U*_eq_(C) for methyl groups and 1.2*U*_eq_(C) otherwise.

## Supplementary Material

Crystal structure: contains datablock(s) I. DOI: 10.1107/S2414314623006399/bv4049sup1.cif

Structure factors: contains datablock(s) I. DOI: 10.1107/S2414314623006399/bv4049Isup2.hkl

Supporting information file. DOI: 10.1107/S2414314623006399/bv4049Isup3.png

Supporting information file. DOI: 10.1107/S2414314623006399/bv4049Isup4.mol

Supporting information file. DOI: 10.1107/S2414314623006399/bv4049Isup5.cml

CCDC reference: 2283625

Additional supporting information:  crystallographic information; 3D view; checkCIF report

## Figures and Tables

**Figure 3:**
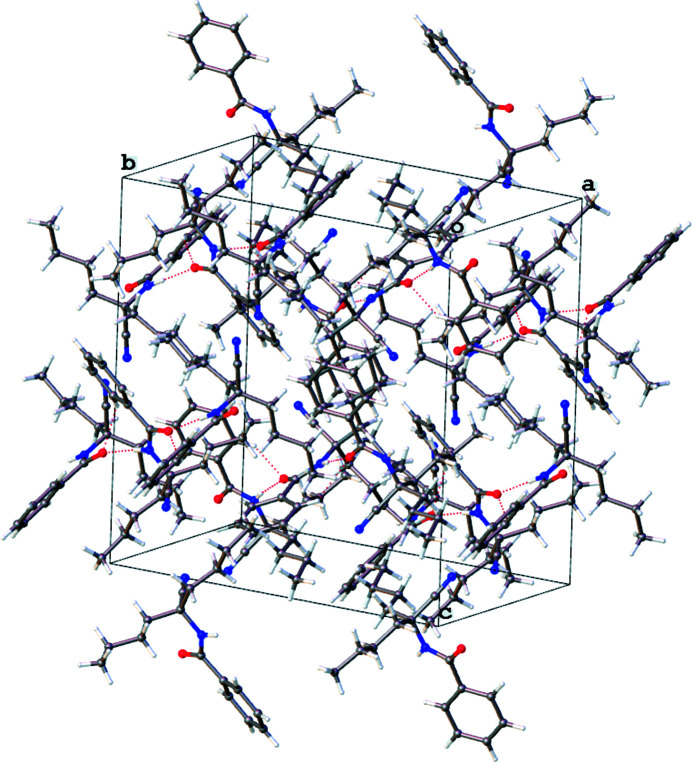
The crystal packing of the title compound. Hydrogen bonds are shown as dashed lines.

**Table 1:** Hydrogen-bond geometry (Å, °)

*D*—H⋯*A*	*D*—H	H⋯*A*	*D*⋯*A*	*D*—H⋯*A*
C7—H7⋯O1^i^	0.93	2.52	3.3046 (17)	142
N2—H2*N*⋯O1^i^	0.860 (16)	2.229 (16)	3.0829 (13)	171.7 (13)

Symmetry code: (i) 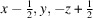 .

**Table 2:** Experimental details

Crystal data	
Chemical formula	C_17_H_24_N_2_O
*M*_r_	272.38
Crystal system, space group	Orthorhombic, *P* *b* *c* *a*
Temperature (K)	298
*a*, *b*, *c* (Å)	10.3939 (1), 17.6680 (2), 17.6653 (2)
*V* (Å^3^)	3244.05 (6)
*Z*	8
Radiation type	Cu *K*α
μ (mm^−1^)	0.54
Crystal size (mm)	0.06 × 0.03 × 0.02

Data collection	
Diffractometer	Rigaku XtaLAB Synergy diffrac­tometer with a HyPix detector
Absorption correction	Multi-scan (*CrysAlis PRO*; Rigaku OD, 2023 ▸)
*T*_min_, *T*_max_	0.852, 1.000
No. of measured, independent and observed [*I* > 2σ(*I*)] reflections	15515, 3322, 2883
*R*_int_	0.027
(sin θ/λ)_max_ (Å^−1^)	0.634

Refinement	
*R*[*F* ^2^ > 2σ(*F* ^2^)], *wR*(*F* ^2^), *S*	0.043, 0.112, 1.08
No. of reflections	3322
No. of parameters	188
H-atom treatment	H atoms treated by a mixture of independent and constrained refinement
Δρ_max_, Δρ_min_ (e Å^−3^)	0.24, −0.19

Computer programs: *CrysAlis PRO* (Rigaku OD, 2023 ▸), *SHELXT* (Sheldrick, 2015*a* ▸), *SHELXL2018* (Sheldrick, 2015*b* ▸), and *OLEX2* (Dolomanov *et al.*, 2009 ▸).

## full crystallographic data

### Crystal data

**Table:**

C_17_H_24_N_2_O	*D*_x_ = 1.115 Mg m^−^^3^
*M**_r_* = 272.38	Cu *K*α radiation, λ = 1.54184 Å
Orthorhombic, *P**b**c**a*	Cell parameters from 10946 reflections
*a* = 10.3939 (1) Å	θ = 4.3–77.8°
*b* = 17.6680 (2) Å	µ = 0.54 mm^−^^1^
*c* = 17.6653 (2) Å	*T* = 298 K
*V* = 3244.05 (6) Å^3^	Needle, clear light colourless
*Z* = 8	0.06 × 0.03 × 0.02 mm
*F*(000) = 1184	

### Data collection

**Table:**

XtaLAB Synergy, Single source at home/near, HyPix diffractometer	3322 independent reflections
Radiation source: micro-focus sealed X-ray tube	2883 reflections with *I* > 2σ(*I*)
Detector resolution: 10.0000 pixels mm^-1^	*R*_int_ = 0.027
ω scans	θ_max_ = 78.0°, θ_min_ = 5.0°
Absorption correction: multi-scan (CrysAlis PRO; Rigaku OD, 2023)	*h* = −10→12
*T*_min_ = 0.852, *T*_max_ = 1.000	*k* = −17→22
15515 measured reflections	*l* = −22→22

### Refinement

**Table:**

Refinement on *F*^2^	Secondary atom site location: difference Fourier map
Least-squares matrix: full	Hydrogen site location: mixed
*R*[*F*^2^ > 2σ(*F*^2^)] = 0.043	H atoms treated by a mixture of independent and constrained refinement
*wR*(*F*^2^) = 0.112	*w* = 1/[σ^2^(*F*_o_^2^) + (0.0401*P*)^2^ + 1.052*P*] where *P* = (*F*_o_^2^ + 2*F*_c_^2^)/3
*S* = 1.08	(Δ/σ)_max_ < 0.001
3322 reflections	Δρ_max_ = 0.24 e Å^−^^3^
188 parameters	Δρ_min_ = −0.19 e Å^−^^3^
0 restraints	Extinction correction: SHELXL2018 (Sheldrick 2015b), Fc^*^=kFc[1+0.001xFc^2^λ^3^/sin(2θ)]^-1/4^
Primary atom site location: dual	Extinction coefficient: 0.00252 (16)

### Special details

**Table:**

Geometry. All e.s.d.'s (except the e.s.d. in the dihedral angle between two l.s. planes) are estimated using the full covariance matrix. The cell e.s.d.'s are taken into account individually in the estimation of e.s.d.'s in distances, angles and torsion angles; correlations between e.s.d.'s in cell parameters are only used when they are defined by crystal symmetry. An approximate (isotropic) treatment of cell e.s.d.'s is used for estimating e.s.d.'s involving l.s. planes.

### Fractional atomic coordinates and isotropic or equivalent isotropic displacement parameters (Å^2^)

**Table:**

	*x*	*y*	*z*	*U*_iso_*/*U*_eq_	
O1	0.68510 (9)	0.39486 (6)	0.24909 (6)	0.0447 (3)	
N2	0.47630 (10)	0.42156 (6)	0.27067 (6)	0.0354 (3)	
H2N	0.3976 (15)	0.4095 (8)	0.2635 (9)	0.039 (4)*	
N3	0.63961 (13)	0.41287 (9)	0.43755 (8)	0.0585 (4)	
C1	0.57087 (12)	0.38404 (7)	0.23375 (7)	0.0346 (3)	
C2	0.53211 (12)	0.32919 (7)	0.17351 (7)	0.0370 (3)	
C3	0.62231 (15)	0.27549 (8)	0.15148 (9)	0.0458 (3)	
H3	0.702073	0.273870	0.175283	0.055*	
C4	0.59447 (18)	0.22447 (8)	0.09449 (10)	0.0563 (4)	
H4	0.655468	0.188838	0.079870	0.068*	
C5	0.4763 (2)	0.22650 (9)	0.05942 (10)	0.0629 (5)	
H5	0.457268	0.191991	0.021279	0.075*	
C6	0.38608 (18)	0.27953 (10)	0.08067 (10)	0.0618 (4)	
H6	0.306382	0.280710	0.056764	0.074*	
C7	0.41359 (14)	0.33116 (9)	0.13756 (9)	0.0485 (4)	
H7	0.352624	0.367046	0.151570	0.058*	
C8	0.13369 (18)	0.42176 (12)	0.51281 (11)	0.0687 (5)	
H8A	0.130708	0.369920	0.496635	0.103*	
H8B	0.048757	0.438170	0.526484	0.103*	
H8C	0.189651	0.426219	0.555814	0.103*	
C9	0.18399 (14)	0.47054 (9)	0.44905 (9)	0.0487 (4)	
H9A	0.190157	0.522524	0.466342	0.058*	
H9B	0.123469	0.469132	0.407272	0.058*	
C10	0.31508 (13)	0.44461 (8)	0.42125 (8)	0.0431 (3)	
H10A	0.371536	0.437325	0.464367	0.052*	
H10B	0.306043	0.396317	0.395690	0.052*	
C11	0.37556 (13)	0.50125 (7)	0.36746 (8)	0.0388 (3)	
H11A	0.390117	0.547983	0.394978	0.047*	
H11B	0.314076	0.512110	0.327604	0.047*	
C12	0.50346 (12)	0.47737 (7)	0.33020 (7)	0.0356 (3)	
C13	0.57641 (13)	0.54701 (8)	0.29944 (8)	0.0433 (3)	
H13A	0.595421	0.580531	0.341460	0.052*	
H13B	0.657822	0.530128	0.278570	0.052*	
C14	0.50566 (16)	0.59173 (8)	0.23929 (9)	0.0510 (4)	
H14A	0.424038	0.608940	0.259590	0.061*	
H14B	0.487752	0.558979	0.196493	0.061*	
C15	0.58252 (18)	0.65952 (9)	0.21246 (11)	0.0601 (4)	
H15A	0.604988	0.690289	0.255974	0.072*	
H15B	0.661983	0.641748	0.189802	0.072*	
C16	0.5124 (2)	0.70834 (11)	0.15573 (11)	0.0803 (6)	
H16A	0.504894	0.681592	0.108633	0.120*	
H16B	0.559606	0.754376	0.147875	0.120*	
H16C	0.428131	0.720084	0.174612	0.120*	
C17	0.58394 (13)	0.44031 (8)	0.38925 (8)	0.0413 (3)	

### Atomic displacement parameters (Å^2^)

**Table:**

	*U*^11^	*U*^22^	*U*^33^	*U*^12^	*U*^13^	*U*^23^
O1	0.0285 (5)	0.0569 (6)	0.0487 (5)	0.0014 (4)	0.0003 (4)	−0.0047 (5)
N2	0.0269 (5)	0.0385 (6)	0.0409 (6)	−0.0016 (4)	0.0001 (4)	−0.0063 (4)
N3	0.0533 (8)	0.0720 (9)	0.0503 (7)	0.0081 (7)	−0.0086 (6)	−0.0002 (6)
C1	0.0304 (6)	0.0375 (6)	0.0359 (6)	0.0015 (5)	0.0029 (5)	0.0027 (5)
C2	0.0373 (7)	0.0361 (6)	0.0377 (6)	0.0003 (5)	0.0048 (5)	0.0007 (5)
C3	0.0473 (8)	0.0395 (7)	0.0506 (8)	0.0057 (6)	0.0068 (6)	0.0012 (6)
C4	0.0712 (11)	0.0392 (7)	0.0584 (9)	0.0067 (7)	0.0142 (8)	−0.0052 (7)
C5	0.0875 (13)	0.0489 (9)	0.0523 (9)	−0.0082 (8)	0.0043 (9)	−0.0148 (7)
C6	0.0600 (10)	0.0696 (11)	0.0558 (9)	−0.0041 (8)	−0.0106 (8)	−0.0160 (8)
C7	0.0430 (8)	0.0544 (8)	0.0482 (8)	0.0044 (6)	−0.0027 (6)	−0.0104 (6)
C8	0.0595 (10)	0.0813 (12)	0.0653 (11)	−0.0059 (9)	0.0233 (9)	0.0014 (9)
C9	0.0400 (8)	0.0557 (8)	0.0505 (8)	−0.0011 (6)	0.0079 (6)	−0.0062 (7)
C10	0.0412 (7)	0.0439 (7)	0.0442 (7)	0.0006 (6)	0.0068 (6)	−0.0020 (6)
C11	0.0358 (7)	0.0384 (7)	0.0422 (7)	0.0023 (5)	0.0031 (5)	−0.0042 (5)
C12	0.0314 (6)	0.0374 (6)	0.0380 (6)	−0.0012 (5)	0.0000 (5)	−0.0041 (5)
C13	0.0384 (7)	0.0405 (7)	0.0509 (8)	−0.0066 (6)	0.0051 (6)	−0.0052 (6)
C14	0.0580 (9)	0.0457 (8)	0.0491 (8)	−0.0105 (7)	0.0018 (7)	0.0016 (6)
C15	0.0675 (11)	0.0460 (8)	0.0668 (10)	−0.0092 (8)	0.0102 (9)	0.0051 (7)
C16	0.1201 (18)	0.0588 (11)	0.0620 (11)	−0.0232 (11)	−0.0118 (12)	0.0109 (9)
C17	0.0350 (7)	0.0459 (7)	0.0428 (7)	−0.0001 (6)	−0.0003 (6)	−0.0064 (6)

### Geometric parameters (Å, º)

**Table:**

O1—C1	1.2327 (15)	C9—H9A	0.9700
N2—C1	1.3531 (16)	C9—H9B	0.9700
N2—C12	1.4691 (16)	C10—C11	1.5164 (19)
N2—H2N	0.855 (16)	C10—H10A	0.9700
N3—C17	1.1393 (19)	C10—H10B	0.9700
C1—C2	1.4946 (18)	C11—C12	1.5422 (17)
C2—C7	1.386 (2)	C11—H11A	0.9700
C2—C3	1.3894 (19)	C11—H11B	0.9700
C3—C4	1.382 (2)	C12—C17	1.4888 (19)
C3—H3	0.9300	C12—C13	1.5439 (18)
C4—C5	1.376 (3)	C13—C14	1.515 (2)
C4—H4	0.9300	C13—H13A	0.9700
C5—C6	1.377 (3)	C13—H13B	0.9700
C5—H5	0.9300	C14—C15	1.516 (2)
C6—C7	1.387 (2)	C14—H14A	0.9700
C6—H6	0.9300	C14—H14B	0.9700
C7—H7	0.9300	C15—C16	1.510 (3)
C8—C9	1.512 (2)	C15—H15A	0.9700
C8—H8A	0.9600	C15—H15B	0.9700
C8—H8B	0.9600	C16—H16A	0.9600
C8—H8C	0.9600	C16—H16B	0.9600
C9—C10	1.5191 (19)	C16—H16C	0.9600
			
C1—N2—C12	122.30 (11)	C9—C10—H10B	109.2
C1—N2—H2N	120.1 (10)	H10A—C10—H10B	107.9
C12—N2—H2N	117.2 (10)	C10—C11—C12	116.38 (11)
O1—C1—N2	121.18 (12)	C10—C11—H11A	108.2
O1—C1—C2	121.11 (11)	C12—C11—H11A	108.2
N2—C1—C2	117.71 (11)	C10—C11—H11B	108.2
C7—C2—C3	119.23 (13)	C12—C11—H11B	108.2
C7—C2—C1	123.33 (12)	H11A—C11—H11B	107.3
C3—C2—C1	117.41 (12)	N2—C12—C17	108.31 (10)
C4—C3—C2	120.54 (15)	N2—C12—C11	108.88 (10)
C4—C3—H3	119.7	C17—C12—C11	107.80 (11)
C2—C3—H3	119.7	N2—C12—C13	112.17 (11)
C5—C4—C3	119.85 (15)	C17—C12—C13	108.73 (11)
C5—C4—H4	120.1	C11—C12—C13	110.83 (10)
C3—C4—H4	120.1	C14—C13—C12	115.10 (11)
C4—C5—C6	120.18 (15)	C14—C13—H13A	108.5
C4—C5—H5	119.9	C12—C13—H13A	108.5
C6—C5—H5	119.9	C14—C13—H13B	108.5
C5—C6—C7	120.29 (16)	C12—C13—H13B	108.5
C5—C6—H6	119.9	H13A—C13—H13B	107.5
C7—C6—H6	119.9	C13—C14—C15	112.07 (14)
C2—C7—C6	119.91 (14)	C13—C14—H14A	109.2
C2—C7—H7	120.0	C15—C14—H14A	109.2
C6—C7—H7	120.0	C13—C14—H14B	109.2
C9—C8—H8A	109.5	C15—C14—H14B	109.2
C9—C8—H8B	109.5	H14A—C14—H14B	107.9
H8A—C8—H8B	109.5	C16—C15—C14	113.86 (16)
C9—C8—H8C	109.5	C16—C15—H15A	108.8
H8A—C8—H8C	109.5	C14—C15—H15A	108.8
H8B—C8—H8C	109.5	C16—C15—H15B	108.8
C8—C9—C10	112.28 (14)	C14—C15—H15B	108.8
C8—C9—H9A	109.1	H15A—C15—H15B	107.7
C10—C9—H9A	109.1	C15—C16—H16A	109.5
C8—C9—H9B	109.1	C15—C16—H16B	109.5
C10—C9—H9B	109.1	H16A—C16—H16B	109.5
H9A—C9—H9B	107.9	C15—C16—H16C	109.5
C11—C10—C9	112.05 (12)	H16A—C16—H16C	109.5
C11—C10—H10A	109.2	H16B—C16—H16C	109.5
C9—C10—H10A	109.2	N3—C17—C12	175.77 (15)
C11—C10—H10B	109.2		
			
C12—N2—C1—O1	0.53 (19)	C8—C9—C10—C11	−169.86 (13)
C12—N2—C1—C2	−179.26 (11)	C9—C10—C11—C12	−174.73 (12)
O1—C1—C2—C7	−159.19 (14)	C1—N2—C12—C17	−55.56 (15)
N2—C1—C2—C7	20.60 (19)	C1—N2—C12—C11	−172.52 (11)
O1—C1—C2—C3	18.64 (18)	C1—N2—C12—C13	64.45 (15)
N2—C1—C2—C3	−161.57 (12)	C10—C11—C12—N2	75.12 (14)
C7—C2—C3—C4	−0.1 (2)	C10—C11—C12—C17	−42.17 (15)
C1—C2—C3—C4	−178.02 (13)	C10—C11—C12—C13	−161.06 (12)
C2—C3—C4—C5	−0.3 (2)	N2—C12—C13—C14	61.10 (15)
C3—C4—C5—C6	0.4 (3)	C17—C12—C13—C14	−179.14 (12)
C4—C5—C6—C7	−0.1 (3)	C11—C12—C13—C14	−60.82 (15)
C3—C2—C7—C6	0.4 (2)	C12—C13—C14—C15	179.43 (13)
C1—C2—C7—C6	178.21 (14)	C13—C14—C15—C16	−176.64 (15)
C5—C6—C7—C2	−0.3 (3)		

### Hydrogen-bond geometry (Å, º)

**Table:**

*D*—H···*A*	*D*—H	H···*A*	*D*···*A*	*D*—H···*A*
C7—H7···O1^i^	0.93	2.52	3.3046 (17)	142
N2—H2*N*···O1^i^	0.860 (16)	2.229 (16)	3.0829 (13)	171.7 (13)

Symmetry code: (i) *x*−1/2, *y*, −*z*+1/2.
