# Supplementary figures and images for: N-(5-Cyano­nonan-5-yl)benzamide
Source: IUCrdata. 2023 Jul 28;8(Pt 7):x230639. doi: 10.1107/S2414314623006399 (PMC10626610; doi:10.1107/S2414314623006399)

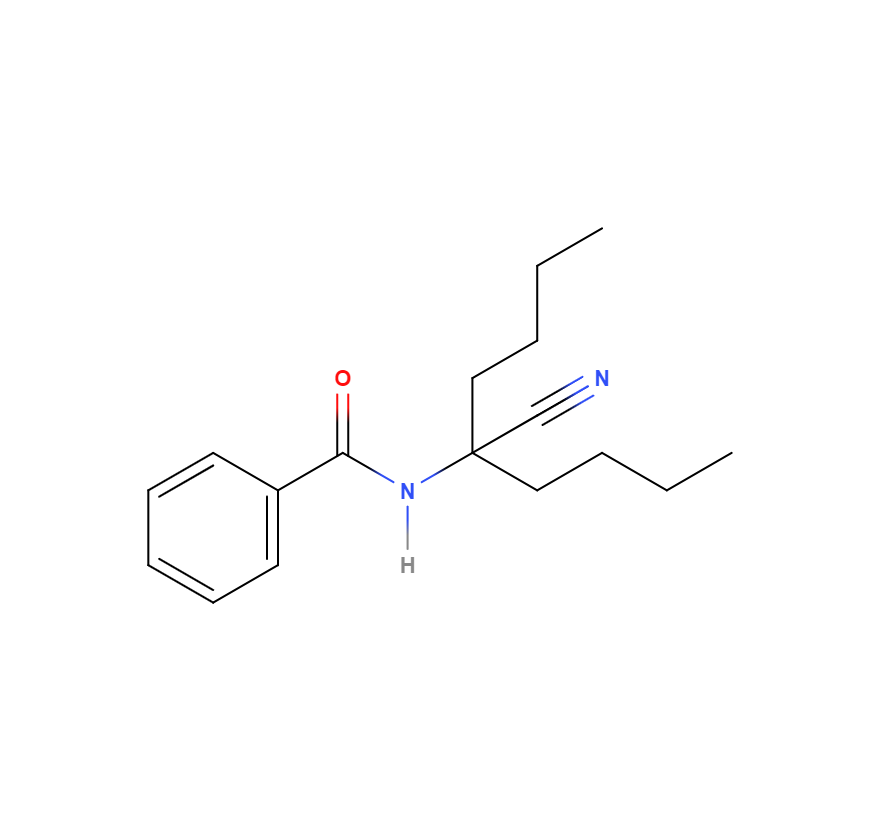

Supplement: Supplementary file 3 [file x-08-x230639-Isup3.png]
